# Supplementary material for: Are carbon emissions trading and green financial instruments synergistic? -Comprehensive quantitative research based on content analysis
Source: PLoS One. 2024 Mar 7;19(3):e0298601. doi: 10.1371/journal.pone.0298601 (PMC10919601; doi:10.1371/journal.pone.0298601)
Supplement: S1 Data — (ZIP) [file pone.0298601.s001.zip › Data/地级市Z_F工作报告.zip!/║╙─╧/╨┬╧τ╩╨/└┤╫╘╣1⁄2╓┌║┼í╛┬φ┐╦╩2╛▌═°í┐_2003.docx]

各位代表：

现在，我代表市人民政府向大会作工作报告，请予审议，并请各位政协委员和其他列席人士提出意见。

一、二○○二年政府工作回顾

2002年，我们在市委的领导下，以“三个代表”重要思想为指导，认真贯彻落实党的十六大精神和省委、省政府的一系列重大部署，坚持以项目统领经济工作全局，团结带领全市人民开拓进取，扎实工作，较好地完成了市九届人大五次会议提出的各项目标任务。

国民经济增速加快，运行质量明显提高。预计全市完成国内生产总值339.5亿元,增长10%。其中第一、二、三产业分别增长1.8%、15.1%、9.3%。规模以上工业企业完成增加值、实现利润分别增长11.3%和40.4%。工业综合效益指数同比提高7.8个百分点。地方财政一般预算收入突破13亿大关，达13.1亿元，增长27.6%。金融机构新增贷款35.62亿元，同比增加16.6亿元；省银企重点项目推介会达成贷款承诺和合同项目150个计98亿元，已履约27.1亿元。全社会用电量增长10.7%，其中工业用电量增长10.3%。社会消费品零售总额增长10.5%。物价指数提高1.2个百分点。

经济结构调整成效突出，重点项目扎实推进。一、二、三产业的比例调整为21.6∶43.7∶34.7,第二产业增加值较上年提高1.8个百分点。全市工业总投资完成18亿元，其中实施千万元以上技改项目38项，完成投资12亿元。新乡火电厂2×13.5万千瓦1号机组、白鹭集团年产3000吨粘胶长丝等14个项目建成投产。高新技术产业增加值占工业增加值的比重提高2个百分点。优质粮和优质畜产品生产加工基地建设取得突破性进展，优质小麦种植面积占麦播面积的72％,61家畜牧、蔬菜生产基地和加工企业获准进京供沪销售。全年奶类总产量增长30%。完成退耕还林5.6万亩。民营企业完成增加值、实现利润均增长15%。电信业新增宽带端口5万个，发展“家庭上网工程”用户近21万户。全社会货物周转量增长6.7%。财产和人寿保险投保收入分别增长11%和79%。旅游门票收入增长82.5%。

固定资产投资快速增长,交通和城市建设创历史新高。全社会固定资产投资98.8亿元，增长34.9%，高于全省平均增速22.7个百分点。全年实施千万元以上基本建设和技改项目186个，总投资55.4亿元，其中国债项目18个，总投资2.7亿元。一是全年完成公路投资21.2亿元，其中地方道路投资15亿元，增长302.8%；新增干线公路 360公里 、县乡公路 180公里 ，新增“村村通”行政村237个。二是完成城建投资13.4亿元，对东干道、六个出市口等 38公里 城区道路进行拓宽改造，对17条主干道两侧579处破旧建筑实施综合开发。三是住宅、市政基础设施等工程全面推进。商品房竣工面积45万平方米；卫河清淤截污等20余项市政工程基本完工；新区行政综合楼等项目开工建设；新增公共绿地 8万平方米 。四是农业基础设施建设得到加强。陈家院水库除险加固等工程进展顺利；二期农网改造已完成93%。

各项改革稳步推进，对外开放取得新成绩。农村税费改革、粮食购销方式和电价改革成效显著，三项合计使农民增收3.3亿元。政府机构改革稳妥进行，市县两级党政机构分别精简28.8%和9.7%，行政编制人数精简24.6%。行政审批制度改革进展顺利，市级行政审批事项精简70%。企业改制稳步实施，华兰公司上市已上报中国证监会，国有企业政策性破产有效推进。财政、金融等项改革进一步深化。外贸出口完成1.41亿美元，增长61.4%；合同利用外资6553万美元，同比增加5427万美元；实际利用外资3693万美元。

社会保障得到加强，人民生活继续改善。“三条保障线”和“两个确保”得到较好落实,争取上级转移支付和市里筹集资金9.51亿元,使56.6万人受益。全市养老、失业保险金和下岗职工基本生活保障金发放率均达到100％；全市享受低保人数89859人,低保覆盖面比上年提高了4个百分点，基本做到应保尽保。帮扶城镇弱势群体和再就业工作成效明显,为1059户颁发了帮扶证，9083人享受到医疗、教育和法律援助；1000名“4050”人员得到安置,25602名下岗职工实现了再就业。全年共发放救济款629万元,保证了灾区群众的基本生活；多渠道争取和安排扶贫资金7996万元,使3.72万人实现脱贫。城镇居民人均可支配收入增长9.7%；农民人均纯收入增长3.3%。

科技、教育等各项社会事业全面发展。全市共有64个项目列入国家和省科技发展计划；新飞、华兰两个 国家级 博士后流动站获准建立；获省科技进步奖8项，居全省第二位；获公开和授权专利累计达到2130项，居全省第三位。农村教育管理体制进一步完善，教师工资由县统一发放。学校布局调整和教师聘任制改革取得新进展。广播电视人口综合覆盖率达93%。参加省九运会金牌总数居全省第五位。人口自然增长率5‰。土地经营收益比上年有大幅度提高。全年关闭规模小、污染严重的制浆造纸企业85家。审计、统计、物价、气象、外事、侨务、地方史志、民族宗教、人防等各项事业都取得了新的成绩。

社会主义精神文明和民主法制建设进一步加强。“三个代表”学习教育活动有效推进。广泛开展“五城创建”、“道德规范进万家”等精神文明创建活动,获得省级“卫生城市”、“园林城市”称号。坚持依法行政,厂务、政务公开取得明显成效。认真执行人大及其常委会通过的决定和决议,积极组织政府职能部门向人大述职,自觉接受政协民主监督。廉政建设和反腐败斗争不断深入。试行“企业安静工作日”制度,开展建设“信用新乡”活动,大力查处涉企“四乱”案件。认真落实信访工作责任制和“一岗双责”领导负责制。社会治安综合治理进一步加强,社会大局稳定。

建立责任、有序、高效的政府运行机制取得进展。坚持行政首长负责制,落实首问负责制、服务承诺制和限时办结制。对公路征地备土、秸杆禁烧、植树造林等重点难点工作实施效能监察,取得明显成效。政府工作人员作风不断转变,工作效率有所提高,真抓实干的工作机制进一步完善。

各位代表，以上成绩的取得，是省委、省政府和市委正确领导的结果，是市人大、市政协大力支持的结果，是全市上下团结奋进、共同努力的结果，得益于多年来新乡经济稳扎稳打、不断整合所奠定的坚实基础，得益于我们有一支过硬的干部队伍，一个政通人和、干事创业的良好氛围。我代表市人民政府向为新乡经济和社会发展做出贡献的全市工人、农民、知识分子、干部、驻新解放军、武警官兵、公安干警及社会各界人士致以崇高的敬意！向关心、支持我市现代化建设的港澳台同胞、海外侨胞和国际友人表示衷心的感谢！

在肯定成绩的同时,我们也必须清醒地看到，我市的经济发展中还存在不少矛盾和问题。国有企业改制滞后，一些国有和乡镇企业生产经营困难；农业结构调整进展不平衡，农民增收难度较大；社会保障能力不强，就业和再就业压力增大;一些县（市）经济增长较慢，财政收支矛盾突出;经济发展环境有待进一步改善;干部队伍中作风不实、效率不高、主动服务意识不强等问题仍不同程度地存在。对此,我们一定要高度重视,在今后的工作中着力加以解决。

二、二○○三年政府工作的指导思想和奋斗目标

按照十六大提出的全面建设小康社会的要求,我市到2020年人均GDP翻两番,总量必须达到1297亿元,年均递增8%以上。按接近或达到2020年全国人均GDP的平均水平计算，总量应达到1536亿元,需要在2000年基础上翻2.4番，年均递增8.9%以上。而且随着基数增大,保持这一速度的难度也会相应增大。根据到2020年非农产业增加值占90%以上、非农就业人口占60%以上、城镇居住人口达到50%以上的目标测算,我市2020年的第二、三产业增加值必须达到1382亿元,年均递增9.8%以上；新增非农就业人口221万人,年均需要安置11万人；城镇居住人口应达到310万人，年均增长8万人，任务非常艰巨。实现这些目标，最根本的是坚持以经济建设为中心，强力推进工业化和城镇化进程，确保经济持续增长和社会全面进步。

今年政府工作的指导思想是：高举邓小平理论伟大旗帜，全面贯彻“三个代表”重要思想，认真落实党的十六大精神和市八次党代会要求，以全面建设小康社会统揽政府工作全局，扩大内需，调整结构，深化改革，扩大开放，优化环境，保持稳定。加快工业化、城镇化、信息化进程，大力推进农业产业化、民营经济规模化、服务业现代化进程，健全完善社会保障体系，发展各项社会事业，努力实现新乡经济全面提速、社会全面进步。

全市经济和社会发展的主要预期目标是：国内生产总值增长10%以上。其中一、二、三产业分别增长4%、13%、10%；全社会固定资产投资增长18%以上；社会消费品零售总额增长10%；外贸出口增长12%以上；实际利用外资增长15%以上；地方财政一般预算收入和GDP同步增长；城镇居民人均可支配收入增长8%，农民人均纯收入增长5%；人口自然增长率控制在7‰以内；城镇登记失业率低于4.5%。

实现上述目标，既是积极可行的，又需要付出艰苦努力。我们要正视前进道路上的困难和问题，更要看到许多有利条件。经济全球化引发的新一轮产业梯度转移，给我们带来了难得机遇；国家继续实施扩大内需的方针,有利于拉动投资和消费需求；实施开放带动战略和西部大开发，有利于我市发挥比较优势和区位优势；周边地市竞相发展，极大地激发了全市人民加快现代化建设的积极性；我市经济快速增长态势和干事创业氛围的形成，为今后新乡经济社会发展创造了良好条件。只要我们抓住机遇，扎实工作，就一定能够实现预期目标，为全面建设小康社会打下坚实基础。

三、以工业化城镇化为重点

强化项目带动 推动经济快速发展

（一）加快工业园区建设，培育骨干企业，推进新型工业化进程

工业化是增强综合经济实力、增加财政收入、提高人民群众生活水平的战略抉择和必由之路。要以高新技术产业为核心，加快产品产业结构调整步伐；以工业园区建设为载体，努力提升工业规模和层次；以重点项目为支撑，做大做强产业群体；以信息化带动工业化，以工业化促进信息化，走科技含量高、经济效益好、资源消耗低、环境污染少、人力资源优势得到充分发挥的新型工业化道路。

培育高新技术产业基地，推进产业结构调整。以新飞、安美、华兰、金龙、环宇、飘安等企业为中心，重点建设白色家电、数字视听、生物制药、铜管材、环保电源、卫材产品生产基地，培育高新技术产业，推进结构调整。加快精密铜管、机械制造、生物制药等工程技术研究中心建设，促进技术创新。以技术改造和整合规范为重点，改造提升机械、纺织、造纸、水泥等重点传统行业，努力提高工业经济整体运行质量和效益。

加快工业园区建设，构建工业发展载体。创新高新技术开发区的生物制药、电子电器等四个专业园区运行机制，力争在招商引资和项目建设方面取得较大突破。积极推进开发区西区和小店工业区基础设施建设，健全机制，完善政策，引导、促进企业入驻发展。抓紧规划建设各县（市）、区的特色工业园区，引导同类产业聚集，逐步形成“成行成市”的专业园区和特色镇。

实施项目带动，培育壮大骨干企业。续建华兰生物公司年产3000万单位冻干人抗凝血酶浓缩物、白鹭化纤集团年产1万吨粘胶长丝二期工程等46个年度投资11.7亿元的重点项目。开工建设新飞电器集团冷藏及保温专用汽车、白鹭化纤公司年产6000吨差别化氨纶纤维等29个年度投资17.2亿元的工业项目及宝泉抽水蓄能电站、豫新发电公司2×30万千瓦热电联产机组等3个基础能源项目。争取开工豫新机械公司大排量汽车空调压缩机生产线技改项目、金龙铜管公司交叉齿高效传热内螺纹铜管生产线技改项目等32个预计年度投资14.7亿元的项目。重点推进安美公司年产60万台背投电视、宝山电厂2×60万千瓦火电机组等35个重大项目的前期工作。力争用3―5年时间，培育5家年销售收入超过30亿元的标志性企业、10家年销售收入超过10亿元的重点企业、20家年销售收入超过5亿元的成长性企业等35家骨干企业，形成一批在国内外市场具有较强竞争力的“拳头”产品，使之成为带动我市工业经济发展的重要支撑力量。

（二）完善城市规划，加快基础设施建设，推进城镇化进程

按照“壮大市区，膨胀县城，发展集镇，整体规划，稳步推进”的原则,以市区为中心城市、长垣县城为副中心城市、各县城为卫星城,沿交通干线发展一批小城镇，构建若干个县域城镇群；完成7个县城和49个建制镇的规划编制；逐步启动23个省市级重点镇的基础设施建设。深化户籍制度改革,完善配套政策,引导农民向城镇合理流动。稳妥推进区划调整,推进城区村委会改居委会,为城市发展奠定基础。

加快路网工程建设。投资4.33亿元续建三原线等9个项目 345.2公里 。投资4.41亿元新开工建设原阳至京珠高速连接线等6个项目 93.7公里 。争取投资9.01亿元开工建设杨小线等9个项目 265.9公里 。切实服务好京珠高速公路建设，力争开工建设新（焦）济高速公路。确保195个行政村实现“村村通”。投资14.1亿元新建改造保健路、化工路等24条 55.3公里 市区道路。

加快实施环保工程。建成投运骆驼湾污水处理厂和第五自来水厂，完成17个水污染治理工程和28个粉尘污染治理项目。加快建设小尚庄污水处理厂。开工建设城市生活垃圾无害化处理场及一批水冲厕所和垃圾中转站。

加快实施绿化亮化美化工程。发动社会力量建设一批小游园和健身休闲景观工程；改造建设牧野公园、卫河公园；启动环城生态绿化工程；加大市区街巷综合整治力度；加快路灯、排水管道等市政配套设施建设。

加快实施住宅工程。续建辉龙花园二期等17项建筑面积178万平方米的住宅工程，新开工建业花园等15项建筑面积166万平方米的住宅工程，力争当年竣工面积超过50万平方米。强化物业管理，争创省级优秀住宅小区2个。

加快实施河渠治理工程。延伸人民胜利渠和卫河清淤、截污、衬砌、铺装工程，加快两岸综合开发和绿化步伐，逐步建成新的景观带和绿化带。做好共产主义渠综合治理工程前期工作。

加快实施大型公共设施工程。续建新辉立交桥、阳光假日购物广场等15项工程；开工建设新长北线立交桥等16项工程；建设“西气东输”配套工程；积极筹划汽车客运东站改造等15项工程。

加快新区建设步伐。建成行政综合办公大楼；基本完成新区中心区道路、供热、排供水管网等配套基础设施建设；加快推进新区住宅社区、学校、医院等项目建设，力争具备居住、办公的基本条件。

（三）培育农村区域性经济中心,推进农业产业化进程

用工业的理念发展农业，以结构调整为主线，推进标准化建设，提高农业产业化和农村工业化水平。依托重点城镇、龙头企业、农贸市场和高效农业园区规划发展一批由众多企业支撑、聚集辐射作用强、经济生产总量大的区域性经济中心，促进农村经济全面发展。

发展壮大绿色畜牧业。以金鑫饲料公司等企业为龙头，重点建设好无公害生猪、蛋鸡生产基地，黄河滩区、城市近郊奶牛养殖基地和黄河故道区、太行山前肉牛、肉羊养殖基地，力争畜牧业产值增长8%。

大力实施粮食优质化工程。以建设全国优质小麦基地和无公害优质水稻基地为载体，健全产业化经营机制，增强市场竞争力。确保优质小麦种植面积稳定在300万亩，争取优质面粉年综合加工能力达到100万吨。

培育发展高效农业园区。积极推广宏力集团红提葡萄园、长远农业生态园运作模式，引导龙头企业进军农业领域。完善唐庄镇果蔬、史庄镇花卉等农业园区运作模式，因地制宜，规划建设一批有区域特色的高效农业园区。

推进林业产业化进程。充分利用国家林业政策，依托黄河林业公司和新亚纸业集团,完成黄河滩区、故道区造纸林生产基地10.6万亩的年度建设任务；启动建设两市一区25个山区和平原沙区乡镇27万亩经济林果基地；完成 1390公里 通道绿化、20万亩退耕还林和20万亩封山育林任务。

积极组织农村劳务输出。建立健全劳务输出体系，拓宽输出领域，提高组织化程度，推动农民多元就业。重点抓好海运、防腐、建筑等领域的劳务输出。力争全年劳务输出达到50万人。

搞好农业和农村基础设施建设。继续抓好大功灌区节水改造、滩区安全建设等15项续建项目；力争正面水库除险加固、石头庄灌区续建配套等17项工程开工建设。继续实施农业综合开发，建设高标准农田。加大农机推广力度，提高农业机械化水平。尽快完成农网改造二期工程，全面实施县城电网改造。加强县乡道路和基层卫生、文化教育等设施建设，改善农村环境条件。积极做好南水北调、黄河中下游综合治理开发配套项目前期工作。

加大扶贫开发力度。加强基础设施建设，加快科技扶贫步伐，改善贫困地区生产生活条件，提高脱贫致富能力。积极争取上级扶贫资金支持，并切实管好用好扶贫资金，力争3万人稳定脱贫。

（四）壮大提升传统行业，发展新兴行业，推进服务业现代化进程

壮大提升商贸流通业、房地产与市政服务业、金融保险证券业、教育卫生服务业四个传统行业。稳步推进商贸流通企业民营化,扩大对外合作,促进业态升级。重点抓好百货大楼、平原商场等现有大型商场的发展,引进国内外知名商业集团来新合作兴业;改造提升批发零售和餐饮业;推进各类宾馆转制升级;培育发展销售额超亿元的连锁及配送企业。积极稳妥发展网上银行、网上证券等新型金融服务。争取国家开发银行、光大银行等多家金融机构在新设点。

积极发展现代物流业、信息服务业、文化旅游业、中介服务业、社区服务业五个新兴行业。打破区域和行业界限，整合物流资源，力争尽早形成以综合物流为基础、城市配送为支撑的两大物流体系。集中建设小店物流港等3个大型物流集团，培育豫北建材大市场等8个大型批发交易中心和配送中心，启动以新乡二机电公司为依托的豫北汽车城等6个物流企业的改造建设。加快发展电子商务和网上教育培训；继续实施“家庭上网工程”，力争信息服务业增加值增长20%左右。以创建A级景区为重点，加快郭亮－八里沟等景区基础设施建设，力争旅游综合收入增长50%以上。发展各类中介机构，规范提高法律咨询、房地产等中介服务业。

规划建设具有政府管理、政府服务、社会志愿服务、公益性服务、商业性服务五大职能的社区服务中心，完善服务功能。大力发展社区服务业，重点扶持华联社区便利店、家政服务等五个项目的建设，优化社区服务环境。

筹建六个功能服务区。以南二环物流中心和小店物流港为主体建设现代物流区，以平原路西段为主体建设中心商业区，以南干道和东干道交叉口周边为主体建设金融商务区，以科教园区为主体建设文化教育区，以辉县、卫辉、北站为重点建设旅游休闲度假区，以体育休闲广场为主体建设健身服务区。通过六个功能服务区的建设推进服务业市场化、产业化、社会化，提高整体水平。

（五）大力发展特色产业、龙头企业和民营经济园区，推进民营经济规模化进程

进一步研究和落实鼓励、扶持政策，放手发展民营经济，逐步提高非公有制经济在国民经济中的比重。将发展民营经济列入政府工作目标，在市场准入、土地使用、贷款等方面为民营企业创造平等竞争环境。引导乡镇企业加快技术改造和机制创新，增强市场竞争能力。

以特色园区为依托，发展壮大龙头骨干企业。加快新乡县振动设备、辉县能源建材、获嘉化工机械、延津粮油加工等特色民营经济园区建设，特别是抓紧建设长垣起重、卫材等首批省级示范园区，尽快形成一定的产业规模优势。以大型骨干企业为主体，引导组建年销售收入5000万元以上的企业集团16个、年销售收入1亿元以上的企业集团8个、年销售收入5亿元以上的企业集团1个。

引导民营经济调整产业结构。完善政策和信息服务体系,扶持农副产品深加工等科技含量高、资源和劳动密集型产业，压缩高污染、高耗能行业。加快技改项目建设，新上千万元以上扩建技改项目33个，其中5千万元以上13个，促进民营经济持续发展。

发展农村合作经济组织，培育经纪人和营销人员队伍。引导农民发展专业合作社等各类合作经济组织，联合抵御市场风险，提高竞争能力。加强培训，切实提高经纪人和营销人员基本素质和营销水平,力争新发展经纪人16万人。

（六）完善信息基础设施，推进信息化进程

完善基础设施,整合信息资源。建立以各级政府机关内部办公业务网等“三网一库”为基本构架的政务信息化体系,加快“金农”、“金盾”、“税务信息化”、“校校通”和“信用新乡”等信息化工程建设。加快固定和移动电话通信网、数据网扩容,整合、优化网络资源，实现信息资源共用共享。

利用信息技术改造传统产业，增强企业活力。广泛推广应用计算机辅助设计、辅助制造、生产控制等信息技术，促进经济增长方式的转变。组织新飞电器等7家单位申报河南省制造业信息化工程重点示范企业，建立完善企业信息服务体系，逐步实现信息资源开发利用的良性循环。

发挥教育和科研资源优势，发展信息制造业。利用驻市高校和科研院所的优势,建立信息技术研发基地，带动信息产业发展。大力发展安美公司卫星通信设备、恒锐中小企业管理系统等信息制造业，逐步形成我市高新技术产业高地。

四、以优化科教资源为重点

发展各项社会事业 促进社会全面进步

（一）整合教育资源，优化人才结构

坚持把教育放在优先发展的战略地位，全面推进素质教育，提高基础教育水平。实施名校带动战略，扩大优质高中办学规模。继续改造薄弱学校，促进教育布局结构调整。整合职业教育资源,筹建市职教中心和职业技术培训学院。大力推进高校扩建和科教园区建设,支持高校发展，促进专科学校升本。按照达标要求,加快党校基础设施建设。

落实“人才兴市”各项优惠政策，重用现有人才，培养后备人才，创造人尽其才的良好环境。选调一批专业技术人才到高等院校深造，选调一批行政管理人才到沿海发达地区挂职锻炼，继续搞好公务员培训，逐步建立一支数量充足、结构合理的高素质人才队伍。

（二）大力促进科技创新

建立和完善以企业为主体的技术创新体系，引导企业参与科技风险投资。加大知识产权的申报、保护和产业化力度，提高企业核心竞争力。力争新建2家省级工程技术中心和1 家 博士后科研流动站。增加高新技术企业3―5家、省级高新技术产品5―8个、省高新技术产业化重点项目5―8项，逐步提高高新技术产业增加值的比重。完善高新区研发创新、产业聚集、孵化创业和转化辐射功能，培育新的经济增长点。积极开展“农业科技年”活动，加速农业科技进步。

（三）坚持基本国策，促进可持续发展

加强人口与计划生育工作，坚持一把手亲自抓、负总责，落实目标管理责任制，确保人口自然增长率低于全省平均水平。重视老龄工作，提高老龄人口的社会化服务水平。合理开发、节约使用和保护水、土地、矿产、森林等资源，完善资源有偿使用机制，提高资源利用效率。抓好重点区域和行业污染治理，减少污染物排放总量。实施生态家园富民工程，严禁秸杆焚烧，开发利用沼气等再生能源，改善生态环境。

（四）推进文化、体育、卫生等社会事业发展

大力推进文化建设，繁荣文学艺术、广播影视和新闻事业。完成广电二期工程建设。加快市档案馆、史志馆建设步伐。促进基层文化馆、图书馆、群艺馆建设。在市区新建一批体彩健身园，为群众提供更多的体育健身场所。开工建设市中心医院外科大楼、市人民医院综合病房楼和新区医院。推进地震监测指挥中心和人防工程建设，做好气象、地震监测预报。重视统计工作，加强对经济社会运行的监测与研究。加大审计力度，强化审计监督。全面贯彻民族宗教政策，增进民族团结。做好侨务、对台工作，更好地服务对外开放。

五、以体制创新为重点

加快改革开放 为发展提供强大动力

（一）深化改革，创新经济管理体制

深化国有资产管理体制和国有企业改革。按照国家、省的统一部署，健全国有资产管理机构，改革经营体制和管理方式。加快企业改制步伐，继续对国有大中型企业实行规范的股份制改造和公司制改革。在新飞公司、白鹭集团等标志性企业中试行股权期权奖励制度。放开搞活国有中小企业，逐步引导国有资本从竞争领域有序退出。加大兼并破产工作力度，促进破产减债和企业发展。推动新钢集团和安钢集团资产重组。华兰公司争取上半年上市,金龙铜业、科隆公司力争早日进入证监会批复程序。

深化农村各项改革。全面贯彻《土地承包法》，稳定农村承包关系。有条件的地方可按照依法、自愿、有偿的原则试行土地承包经营权流转，逐步发展规模经营。巩固农村税费改革成果，搞好各项配套改革，全面实行涉农经营性收费公示制。深化粮食流通体制改革，探索对粮农直接补贴的办法，保护农民利益。推进农村医疗卫生体制改革，开展合作医疗试点工作。

深化投融资体制改革。以市场为导向，逐步建立责权利明确的投融资风险机制，激活民间投资。完善市建设投资公司、经济技术开发公司、中小企业信用担保中心的法人治理结构，新组建交通道路开发公司、城市建设投资开发公司、科教园区投资发展公司和土地整理开发有限公司，发挥政府投资的导向作用，拓宽投资领域，扩大融资平台。

深化行政管理体制改革。巩固行政机构改革成果，推进事业单位机构改革。创新政府管理经济的工作机制和方式，完善政府重大决策的规则和程序，把政府的决策逐步调整到规划发展战略、优化发展环境、提供优质服务上来。深化行政审批制度改革，压缩审批事项，改进审批方式，努力探索外资企业、重点企业网上申报、审批新办法。

完善财税金融工作运行机制。调整财政支出结构，坚决压缩会议、招待、出国考察、车辆购置、临时公用经费等一般性支出，保证工资发放、社会保障和重点生产性基础设施建设。深化部门预算、会计委派、“收支两条线”、政府采购等财政制度改革，建立申请、审核、拨付、执行、效益评估相结合的跟踪管理机制。坚持依法治税，强化税收征管，确保财政收入与经济同步增长。加大国债项目争取力度，落实地方配套资金，严格国债资金的管理和使用。积极争取金融对地方经济发展的支持，落实金融激励政策，完善中小企业担保服务体系，深化农村金融机构改革，严厉查处逃废金融债务行为，取缔各种非法金融活动，努力创建金融安全区。

清除限制消费的障碍，完善消费政策措施。开放和培育住房二级市场，规范住房租赁市场，加快住房制度改革。积极培育新的消费热点，促进住房、旅游、体育健身、教育等方面的消费。扩大消费信贷，优化消费环境，改进消费服务，力争全社会消费有较大幅度的增长。

（二）实施再就业工程，加强社会保障体系建设

将控制失业率、增加就业岗位作为政府重要职责，纳入工作目标。全面清理下岗职工再就业的各项行政性收费，落实税费减免等扶持政策。加大对就业再就业工作的资金投入。健全就业再就业社区平台，完善市、区、街道三级再就业服务网络。积极开展再就业援助，城市公益性岗位优先安排特困群体就业。

巩固“两个确保”，做好“低保”工作。确保国有企业下岗职工基本生活费、企业离退休人员养老金按时足额发放。规范城市居民最低生活保障工作，合理确定“低保”标准和“低保”对象补助水平，做到应保尽保。搞好“三条保障线”的衔接，积极稳妥地开展下岗职工基本生活保障向失业保险并轨，认真做好城镇各项社会保险的扩面征缴工作。继续大力帮扶弱势群体，妥善解决困难群众在住房、就医、子女入学等方面的实际问题。落实社保资金投入，建立稳定、可靠、多渠道的社会保障筹资体系。

（三）完善外经贸工作体系，推动外向型经济发展

健全市对外开放领导机构，实行联席会议制度，落实各项工作措施，加强沟通合作。完善县（市）、区外经贸工作管理机构和咨询服务体系，抓好外商投诉中心和外商投资服务中心建设。健全对外招商目标管理体系，对市直有关部门下达招商引资任务，落实目标责任制。

创新招商机制。建立社会招聘、全员聘用、年薪浮动和末位淘汰的招商引资工作机制，使招商人员的责、权、利挂钩，奖惩分明。充分发挥企业和各类中介组织招商引资的作用，鼓励有关部门和民间力量利用各种渠道到国内外招商引资。拓宽利用外资领域，吸引外资参与我市国有企业改造。

扩大外经外贸规模。健全外贸出口激励政策，落实外贸出口目标责任制。坚持科技兴贸和以质取胜，优化出口商品结构，积极扩大高新技术产品、服务贸易和农副产品出口规模。培育外贸骨干企业和生产基地，重点扶持巨晶科技公司等一批企业获得产品自营出口权。鼓励有实力、上规模的企业申请境外工程承包和劳务合作经营权，提高对外经济合作的层次和水平，力争新增2家以上企业获得外经经营权。

（四）深化“五城”同创，提高城市现代化水平

围绕“文明城市”、“卫生城市”、“旅游城市”、“园林城市”、“信用城市”的创建目标,明确责任,突出重点,齐抓共管。完善城管工作体系,充分发挥基层组织在城市管理中的主力军作用。加强市容长效管理,建立专业化的执法队伍,制定统一的管理办法,完善考评、督查和奖惩机制，提高城市管理水平。发动群众广泛参与“五城”创建活动，提高市民素质和城市文明程度。

（五）整顿和规范市场经济秩序，优化经济发展环境

依法规范建筑、文化市场和税收征管秩序，严厉打击制假售假、非法传销等违法犯罪活动。加强安全生产，严防各类安全事故发生。打破地方保护和行业垄断,创造市场主体平等竞争的环境。高度重视信用制度建设,加快建立企业和个人信用档案,努力建设“信用新乡”。从严控制检查评比,认真落实企业“安静工作日”制度,集中整治企业经营环境。以公正严明的法制环境、优质高效的服务环境、规范有序的市场环境、统一稳定的政策环境、诚实守信的发展环境、安全文明的社会环境,营造经济发展的“家”环境。

六、以依法治市为重点

推动社会主义政治文明和精神文明健康发展

坚持依法治市，依法行政。自觉接受人大及其常委会的法律监督,认真执行人大及其常委会通过的各项决定和决议。加强与人民政协的协商,接受政协的民主监督。认真办理人大代表建议和政协委员提案,不断提高办理质量。广泛听取各民主党派意见，发挥工、青、妇等人民团体的桥梁纽带作用。健全基层自治组织和民主管理制度，扩大基层民主。

加强法制宣传教育，提高公民法律素质。保护妇女儿童、残疾人和未成年人的合法权益。强化社会治安综合治理，坚决杜绝“黄、赌、毒”等丑恶现象。继续保持“严打”态势，深入开展创建安全城市和“人民满意的政法干警（单位）”活动。依法惩治“法轮功”等邪教组织犯罪活动。高度重视信访工作，继续落实信访工作责任制和“一岗双责”领导负责制，坚持依法信访，认真解决群众反映的问题，正确处理人民内部矛盾，妥善处置各种群体性事件，确保社会大局稳定。大力加强全民国防教育，关心支持军队和国防后备力量建设，深入开展拥军优属和军民共建活动，实现全国“双拥模范城”三连冠。

深入学习贯彻十六大精神和“三个代表”重要思想，切实加强思想政治工作。贯彻公民道德建设实施纲要，广泛进行社会公德、职业道德和家庭美德教育，不断提高人们的思想道德素质。继续开展“热爱新乡，增辉中原”等各类文明创建活动，认真组织科技、文化、卫生三下乡活动。崇尚科学，反对迷信，倡导科学文明健康的生活方式。

大力加强政府自身建设。坚持行政首长负责制,一级对一级负责,一级支持一级工作。牢固树立“便民、高效、廉洁、规范”的服务宗旨，主动为群众服务，为基层服务，为企业服务。健全行政效能监察制度，建立绩效考核机制，落实“上追一级”的责任追究制度，建立责任、有序、高效的工作运行机制。全面落实党风廉政建设责任制,认真纠正部门和行业不正之风,坚决惩处各种以权谋私、腐败堕落的行为,树立政府系统协调、高效、务实、廉洁的良好形象。

牢固树立执政为民的思想,务必保持谦虚、谨慎、不骄、不躁的作风,务必保持艰苦奋斗的作风,把发展作为第一要务,勤奋学习,踏实工作,聚精会神搞建设,一心一意谋发展；牢固树立宗旨意识和公仆意识，倾听群众呼声，体察群众情绪，关心群众疾苦，扎扎实实地帮助群众解决困难，实实在在地为群众谋取利益，不辜负全市人民的期望。

各位代表，新的一年，我们的任务艰巨而光荣。让我们紧密团结在以胡锦涛同志为总书记的党中央周围，高举邓小平理论伟大旗帜，全面贯彻三个代表重要思想，认真落实十六大精神，在省委、省政府和市委的领导下，在市人大、政协的监督支持下，抢抓机遇，开拓创新，同心同德，埋头苦干，全面完成今年经济和社会发展的各项任务，为全面建设小康社会而努力奋斗！
